# Supplementary material for: U.S. CDC support to international SARS-CoV-2 seroprevalence surveys, May 2020–February 2022
Source: PLOS Glob Public Health. 2022 Aug 5;2(8):e0000658. doi: 10.1371/journal.pgph.0000658 (PMC9490761; doi:10.1371/journal.pgph.0000658)
Supplement: Attachment — Submitted filename: R1_Response.docx [file pgph.0000658.s002.docx]

Thanks for your comments and questions. Please find below our responses and clarifications:

- RE: COPYRIGHT of materials and figures
  - Please note that all figures and other content in our paper is developed originally by our team. We do not need to get special permissions from any other team.
  - Figure 1: We developed the map using
    1. QGIS software which is a free and open-source geographic information system
    2. Natural earth layer (public domain)
       - Link: <https://www.naturalearthdata.com/downloads/110m-cultural-vectors/110m-admin-0-countries/>
       - Term of use: <https://www.naturalearthdata.com/about/terms-of-use/>
  - Included this information in the updated version of the manuscript as well.
- RE: Data used
  - Instructions noted. To facilitate the review process, we are submitting the subset of our data that we used for this analysis and paper. Please note that
    1. This is only for facilitating the review process. The data set should not be shared or published publicly.
    2. This is the current data set, which is slightly different from the one we used for this analysis, which stopped by February 28, 2022.
- RE: Live participants and ethical statement
  - Please note that our study does not include live participants. It is just tracking and analyzing other studies that do. Our data is just programmatic; tracking other studies parameters, implementation, and results; and does not include any individual participant data.
  - CDC board has assessed our study and judged it to be non-Research under 45 CFR 46.102(l). ID: 0900f3eb81de998d. Accession #: CGH-GRRT-10/7/21-e998d.
  - We have used the appropriate language as recommended by CDC and the federal government in our paper Line 134.
  - We edited the manuscript following your instructions for clarity.
