# Supplementary material for: U.S. CDC support to international SARS-CoV-2 seroprevalence surveys, May 2020–February 2022
Source: PLOS Glob Public Health. 2022 Aug 5;2(8):e0000658. doi: 10.1371/journal.pgph.0000658 (PMC9490761; doi:10.1371/journal.pgph.0000658)
Supplement: Attachment — Submitted filename: R2_Responses.docx [file pgph.0000658.s003.docx]

Thanks for your comments and questions. Please find below our responses and clarifications in blue:

1. **Journal Requirements:**
   - Please update the 'Competing Interests' statement with this "The authors have declared that no competing interests exist".
     - Done, thanks.
   - Please amend your detailed Financial Disclosure statement. This is published with the article. It must therefore be completed in full sentences and contain the exact wording you wish to be published.

Please clarify all sources of funding (financial or material support) for your study. List the grants (with grant number) or organizations (with url) that supported your study, including funding received from your institution.

State the initials, alongside each funding source, of each author to receive each grant.
State what role the funders took in the study. If the funders had no role in your study, please state: “The funders had no role in study design, data collection and analysis, decision to publish, or preparation of the manuscript.”

If any authors received a salary from any of your funders, please state which authors and which funders.

If you did not receive any funding for this study, please simply state: “The authors received no specific funding for this work."

- The authors received no specific funding for this work.
- Added the statement to the manuscript.
  - In the online submission form, you indicated that “Data shared with reviewers. Not to be published publicly. The authors can consider sharing data upon request.”. All PLOS journals now require all data underlying the findings described in their manuscript to be freely available to other researchers, either: 1. In a public repository, 2. Within the manuscript itself, or 3. Uploaded as supplementary information.

This policy applies to all data except where public deposition would breach compliance with the protocol approved by your research ethics board. If your data cannot be made publicly available for ethical or legal reasons (e.g., public availability would compromise patient privacy), please explain your reasons by return email and your exemption request will be escalated to the editor for approval. Your exemption request will be handled independently and will not hold up the peer review process, but will need to be resolved should your manuscript be accepted for publication. One of the Editorial team will then be in touch if there are any issues.

- Noted, thanks.
- De-identified data was uploaded as supplementary information.
  - Please review your reference list to ensure that it is complete and correct. If you have cited papers that have been retracted, please include the rationale for doing so in the manuscript text, or remove these references and replace them with relevant current references. Any changes to the reference list should be mentioned in the rebuttal letter that accompanies your revised manuscript. If you need to cite a retracted article, indicate the article’s retracted status in the References list and also include a citation and full reference for the retraction notice.
    - Done—no edits added. Thanks.

2. **Reviewers’ comments:**
   - Reviewer #1: The manuscript is technically good. It presents all the mandatory sections for an article and follows the steps of a scientific research. However, the results are interesting for the study funder itself and do not impact knowledge about the object of the study for people outside the organization. Despite being technically flawless, the manuscript is more of a technical report than original research.
     - Thanks for the feedback. Although our results are specific to seroprevalence studies funded by CDC, we do believe they are interesting and helpful to other entities supporting seroprevalence. In addition, in our paper, we also discuss ongoing challenges in implementing seroprevalence studies and interpreting seroprevalence results (introduction of vaccine, cross-reactivity, correlates of protection, etc.); all of which are extremely relevant and timely for other organizations working in seroprevalence.
   - Reviewer #2: the article is very interesting. clearly summarizes the CDC's investment in research on the seroprevalence of covid-19. It missed work in Brazil, since it is one of the countries with the highest number of cases and deaths. But this does not rule out the importance of publication.
     - Thanks for the feedback.
   - Reviewer #3: This paper summarized the CDC’s support of 72 international SARS-CoV-2 seroprevalence studies in 35 low- and middle-income countries. The paper describes the type of support the CDC provided (technical or financial assistance), the geographic distribution of support, study design including laboratory procedures, and population surveyed. The paper was clearly written and describes important ongoing global public health surveillance efforts. The discussion clearly describes the limitations to seroprevalence surveys, including the need for study designs to differentiate between vaccine-induced and naturally acquired immunity. The manuscript was very clear in its description of CDC activities to date.
     - Thanks for the feedback.
   - A few comments:
     - L203. The paper mentions that results from 5 serosurveys supported by the CDC have been published to date and 15 more have been shared with MOH. Please clarify the plans for dissemination of the remaining studies.
       - Thanks for the comment. When data analysis in complete, results are immediately shared with local authority so they can act on it accordingly and in a timely manner. Manuscript publication is generally led by ministry of health and takes longer time to complete. We are currently supporting the process of getting the remaining manuscripts out as soon as possible. This is discussed in the paragraph starting in line 243.
     - Addressing the lack of global access to antibody tests is an urgent global health priority. I would suggest adding a paragraph in the Discussion to discuss access to antibody testing and the glaring need for international support, especially from the US, to improve global equity in access to antibody tests critical for global health surveillance.
       - Thanks for the very important comment. We have added this point to the discussion paragraph on challenges to the implementation of seroprevalence surveys (line 270).
       - Please note that as a US federal agency, CDC is not allowed to use language in its publications that may seem as if we are requesting more funding or support from congress.
     - Additionally, I would suggest discussing the glaring disparities in infection-to-case ratios (Table 2) through a lens of health equity. The high infection-to-case ratios identified in many low- and middle-income countries highlights the critical need for health systems building and improved population surveillance.
       - Thanks for the suggestion. In paragraph starting line 243, we discuss these numbers and possible explanations for these disparities, clarifying that, in addition to weak public health systems in low- and middle-income settings, these variations could also be explained by differences in clinical presentation (less symptomatic) or mitigation measures in these settings.
       - Edited for clarity.

While revising your submission, please upload your figure files to the Preflight Analysis and Conversion Engine (PACE) digital diagnostic tool, <https://pacev2.apexcovantage.com/>. PACE helps ensure that figures meet PLOS requirements. To use PACE, you must first register as a user. Registration is free. Then, login and navigate to the UPLOAD tab, where you will find detailed instructions on how to use the tool. If you encounter any issues or have any questions when using PACE, please email PLOS at [figures@plos.org](mailto:figures@plos.org). Please note that Supporting Information files do not need this step.

- Done, thanks.
